# Meteorological Table, for July 1854

**Published:** 1854-08

**Authors:** Lawrence Young


### METEOROLOGICAL TABLE, FOR JULY 1854.

BY LAWRENCE YOUNG, ESQ.

| Day of month. | Morning. | 2 o'clock, P. M. | Evening. | Mean temperature. | Barometer. | Rain-gauge. | Course of wind. | Remarks. |
|---|---|---|---|---|---|---|---|---|
| 1 | 70 | 94 | 85 | 83 | 29.51 | | n. e. | Variable. |
| 2 | 72 | 94 | 87 | 84 | 29.60 | | e. n. e. | do |
| 3 | 69 | 95 | 83 | 82 | 29.56 | | w. s. w. | do |
| 4 | 73 | 96 | 79 | 83 | 29.52 | | s. | do |
| 5 | 72 | 95 | 77 | 81 | 29.52 | | s. w. | do |
| 6 | 68 | 89 | 81 | 79 | 29 53 | | s. s. w. | do |
| 7 | 71 | 93 | 83 | 82 | 29.57 | 10 | s. | do |
| 8 | 73 | 92 | 77 | 81 | 29.58 | 79 | s. | do |
| 9 | 71 | 74 | 77 | 74 | 29.58 | 16 | n. n. e. | Cloudy. |
| 10 | 67 | 82 | 74 | 74 | 29.63 | | e. n. e. | Clear. |
| 11 | 53 | 83 | 69 | 68 | 29.61 | | n n. e. | do |
| 12 | 59 | 83 | 74 | 72 | 29.61 | | n. | do |
| 13 | 57 | 86 | 77 | 73 | 29.62 | | n. n. w. | Variable. |
| 14 | 55 | 88 | 79 | 74 | 29.56 | | n. e. | Clear. |
| 15 | 57 | 92 | 80 | 77 | 29.61 | | w. | do |
| 16 | 53 | 94 | 84 | 78 | 29.59 | | w. | Variable. |
| 17 | 69 | 97 | 78 | 81 | 29.70 | 12 | s. w. | Cloudy. |
| 18 | 68 | 98 | 84 | 83 | 29.68 | | w. | Clear. |
| 19 | 62 | 98 | 83 | 81 | 29.56 | | n. n. e. | do |
| 20 | 65 | 101 | 88 | 85 | 29.48 | | w. s. w. | do |
| 21 | 69 | 101 | 79 | 83 | 29.50 | 16 | w. s. w. | Variable. |
| 22 | 72 | 98 | 82 | 85 | 29.50 | | w. s. w. | do |
| 23 | 66 | 96 | 86 | 84 | 29.53 | | w. s. w | do |
| 24 | 65 | 97 | 81 | 81 | 29.55 | | w. s. w. | do |
| 25 | 75 | 95 | 86 | 85 | 29.40 | 15 | s. | do |
| 26 | 66 | 77 | 70 | 71 | 29.52 | 12 | n. | do |
| 27 | 54 | 87 | 75 | 72 | 29.71 | | w. | Clear. |
| 28 | 55 | 94 | 81 | 77 | 29.66 | | w. | do |
| 29 | 65 | 96 | 87 | 83 | 29.58 | | w. | do |
| 30 | 67 | 99 | 88 | 84 | 29.63 | | w. s. w. | do |
| 31 | 71 | 99 | 92 | 87 | 29.68 | | w. | do |
| | | | | 79.5 | | 1.60 | | |

